# Effect of mind–body therapies on anxiety, depression and sleep quality in college students: a network meta-analysis

**DOI:** 10.3389/fpubh.2026.1767300

**Published:** 2026-03-19

**Authors:** Dingnan Zhang, Hongchang Yang, Yubo Gao, Shenghua Min

**Affiliations:** 1 Physical Education Department of Hohai University, Nanjing, China; 2 College of Humanities and Social Sciences of Nanjing University of Aeronautics and Astronautics, Nanjing, China

**Keywords:** anxiety, college students, depression, mind–body therapies, sleep quality

## Abstract

**Introduction:**

Mind–body therapies (MBTs) have demonstrated beneficial effects on anxiety, depression, and sleep quality in college students. However, few studies have compared the efficacy of different types of MBTs. Therefore, we conducted this study to compare and rank the relative effects of various MBTs on these outcomes.

**Methods:**

A systematic search was conducted across seven electronic databases (inception-July 8, 2025) for randomized controlled trials investigating the effects of MBTs on depression, anxiety and sleep quality among college students. Literature selection was performed in accordance with the PICOS framework, and data extraction was carried out independently by two reviewers. Risk of bias was assessed via the Cochrane risk of bias tool, version 2 (RoB 2.0) and the Grading of Recommendations Assessment, Development, and Evaluation (GRADE) framework. Both pairwise and network meta-analyses were performed via Stata software, which generated forest, network, and funnel plots. Furthermore, we adopted the surface under the cumulative ranking curve (SUCRA) to evaluate and rank the intervention effects of different MBTs on depression, anxiety and sleep quality among college students.

**Results:**

Twenty-seven eligible studies with 2,664 participants were included. This network meta-analysis revealed significant differences in the impact of MBTs on sleep quality among college students compared with the control group. Baduanjin [SMD = −1.29, 95% CrI (−2.67, −0.02)] and Qigong [SMD = −2.24, 95% CrI (−3.85, −0.44)] demonstrated the most notable effects. Additionally, for anxiety, yoga [SMD = −3.72, 95% CrI (−6.26, −1.47)] was significantly more effective than the control. Furthermore, for depression, Qigong [SMD = −3.9, 95% CrI (−5.76, −1.37)] was significantly better than the control group. Furthermore, Qigong was significantly more effective than yoga [SMD = 3.78, 95% CrI (0.96, 5.99)] and Baduanjin [SMD = 3.35, 95% CrI (0.03, 5.27)]. The SUCRA ranking identified Qigong (SUCRA = 90.5%) as the best intervention for sleep quality, yoga (SUCRA = 89.0%) for anxiety, and Qigong (SUCRA = 95.0%) for depression.

**Conclusion:**

The current evidence indicates that Qigong is the optimal intervention for alleviating depression and improving sleep quality, whereas yoga is the most effective intervention for alleviating anxiety. Nevertheless, due to the limited number of studies included, further research is needed to enhance the reliability of the findings.

**Systematic review registration:**

https://www.crd.york.ac.uk/PROSPERO/view/CRD420251104941, identifier (CRD420251104941).

## 1 Introduction

Anxiety, depression, and sleep disturbances pose significant, intertwined mental health challenges for college students, exerting adverse effects on both their academic trajectories and overall wellbeing. As they transition from adolescence to adulthood, college students represent a pivotal group whose psychological resilience has not yet fully matured; consequently, they confront multiple overlapping challenges pertaining to academic pursuits, identity formation, and social adaptation (1, 2). These transitions are compounded by diverse stressors, such as intense academic competition, disruptions from public health emergencies (e.g., the COVID-19 pandemic), and challenges associated with adapting to independent living (1). These characteristics predispose college students to a high prevalence of combined mental health issues and sleep disturbances. Furthermore, evidence indicates that college students are at high risk for depression (3, 4). These adverse outcomes impair academic performance and quality of life (5), exacerbate psychological distress (6), and may lead to self-destructive tendencies (7). Within this context, a critical contradiction arises: the growing demand for mental health support far outpaces the capacity of existing interventions, which are characterized by a lack of standardized protocols and an inability to meet the large-scale needs of college students (1, 5). This gap highlights the urgent need for safe, accessible, and effective nonpharmacological interventions aimed at addressing the pressing public health challenge of college students’ mental health (5, 8).

Mind–body therapies (MBTs) constitute a category of practices that emphasize the synergistic integration of mental and physical processes. In contrast to traditional physical training, which primarily targets physiological outcomes (e.g., enhancing fitness or muscle strength), MBTs aim to improve overall health through the synchronized regulation of physical and psychological states (9). Predominant MBTs include yoga (e.g., laughter yoga, gentle yoga), tai chi (e.g., the 24-form simplified style), baduanjin, and mindfulness meditation (10). Although these practices vary in expression, they share a common mechanistic pathway: fostering mind–body connections through the synergistic integration of physical control, breathing regulation, and attentional training. This typically involves (i) using specific postures or movements as a foundation (11), (ii) incorporating regulated breathing techniques (12), and (iii) simultaneously conducting focus and emotional regulation training (13), ultimately achieving a synergistic psychophysical balance. Research indicates that MBTs positively impact student mental health, effectively reducing symptoms of depression and anxiety (14) and concurrently improving sleep quality and reducing the risk of sleep disorders (14, 15). The advantages of MBTs—including their nonpharmacological safety, accessibility, low complexity, and minimal facility demands—make them particularly amenable to standardized, large-scale dissemination in universities, effectively addressing current gaps in student health promotion (16) and effectively addressing current gaps in university mental health services owing to their potential for standardization and scalability. However, conventional interventions for student mental health are plagued by substantial limitations. Pharmacological treatments are associated with risks of adverse effects and drug interactions (17), whereas psychological counseling faces challenges such as social stigma (18) and limited resource availability (19). The substantial heterogeneity in intervention protocols—evident in the wide variation of parameters such as session frequency (5–24 sessions) and duration—undermines the comparability of findings across studies (15, 20). A further critical concern pertains to the overall quality of the evidence. Most systematic reviews in this area are rated as having “critically low” or “low” methodological quality (21), and primary studies frequently suffer from a high risk of bias, limited sample sizes, and an absence of long-term follow-up (22). Consequently, this fragmentation and lack of standardization hinder the translation of existing research into practical, actionable guidance for universities (23, 24).

Network meta-analysis (NMA) provides a methodological framework to address this gap. When head-to-head trials comparing multiple interventions are unavailable or insufficient, NMA synthesizes direct and indirect evidence through a common comparator (25). This approach enables the estimation of relative effects among all interventions and allows ranking of their probability of being the most effective, even in the absence of direct comparisons (26). By applying NMA, the present study aims to transcend the limitations of pairwise meta-analyses and offer a comprehensive comparative evaluation of five MBTs for anxiety, depression, and sleep quality in college students.

Although previous studies have demonstrated the positive effects of MBTs in alleviating anxiety and depression and improving sleep quality among college students, the relative effectiveness of different types of MBTs for these three outcomes remains unclear. Thus, conducting a systematic analysis and meta-analysis of randomized controlled trials (RCTs) in this field is necessary. To our knowledge, this gap remains unaddressed in the literature. The scope of this systematic review and NMA is to compare and rank the relative efficacy of five common MBTs (yoga, Qigong, Tai Chi, Baduanjin, and Yijinjing) specifically among college students (aged 18–29 years) for three distinct but interrelated outcomes: anxiety, depression, and sleep quality. By addressing this literature gap, our study seeks to offer valuable guidance for the practical application of MBTs in university settings.

## 2 Materials and methods

### 2.1 Protocol and guidance

This systematic review followed the framework of the Cochrane Handbook for Systematic Reviews of Interventions (27) and the Preferred Reporting Items for Systematic Reviews and Meta-Analysis guidelines (28). The protocol for this review was prospectively registered on PROSPERO on June 3, 2025, and there was no deviation from it (CRD420251104941): https://www.crd.york.ac.uk/PROSPERO/view/CRD420251104941.

### 2.2 Literature search strategy

A thorough review of the literature was carried out by searching seven databases, including the Cochrane Library, PubMed, Embase, Web of Science, Wanfang Data, VIP Database, and China National Knowledge Infrastructure (CNKI), to identify RCTs examining the effects of MBTs on sleep quality, anxiety, and depression among college students, with the search concluding on July 8, 2025. Only studies published in English or Chinese were included. To enhance the comprehensiveness of the search, the key terms included “students,” “mind–body therapies,” “sleep,” “anxiety,” and “depression.” To further expand the search, relevant synonyms were included when the titles, abstracts, and keywords in these databases were searched. (See Supplementary Tables 1–6 for full strategies for the databases.)

### 2.3 Inclusion and exclusion criteria

The study strictly formulated the inclusion and exclusion criteria for the selection of literature, adhering to the PICOS principle (27). The inclusion criteria for the literature were as follows:

1. Population: Eligible participants were college students aged 18–29 years.
2. Intervention: MBTs were administered to the experimental group. Defined by the synergistic integration of voluntary mental focus, controlled breathing techniques, and specific postures or movements, MBTs aim to foster closer mind–body connections, with examples including yoga, Tai Chi, and specific regulated breathing and movement practices often categorized under the broad umbrella of ‘Qigong’ (such as Baduanjin and Yijinjing). For the purpose of this NMA, and due to their distinct, standardized movement sequences reported in the included trials, Baduanjin, Yijinjing, and other Qigong practices (referred to as ‘Qigong’ node) were treated as separate intervention nodes. This allows for a direct comparison of these specific, commonly researched forms. Additionally, no mandatory guidelines were set for the timing, intensity, or duration of the MBTs intervention. No restrictions were applied to blinding, as complete blinding is extremely challenging for exercise-based interventions.
3. Comparisons: Eligible studies were required to include at least two groups (experimental/intervention groups). The control group could either refrain from any exercise intervention and continue with their regular daily activities or receive other types of MBTs.
4. Outcomes: The present study analyzed at least one psychological outcome from the included studies: depression, anxiety, or sleep quality. When multiple follow-up assessments were conducted, the longest assessment was selected.

The exclusion criteria were duplicate publications; animal studies; case reports; conference abstracts; review articles; inaccessible full texts; publications not in English or Chinese; and studies where the intervention arm was combined with other therapies (e.g., pharmacological, dietary, psychological, or educational therapies).

### 2.4 Definition of each MBTs

Qigong is a traditional Chinese mind–body exercise that combines slow, flowing movements, controlled breathing, and focused meditative intention (29), characterized by body movement, spiritual guidance and controlled breathing (30). Yoga is a mind–body practice that originates from ancient Indian philosophy and integrates physical postures, regulated breathing, and meditation or mindfulness techniques (31). Tai Chi is a martial-art-derived mind–body practice characterized by slow, continuous, and deliberate circular movements, weight shifting, and coordinated breathing. It is often described as “meditation in motion” and requires sustained attention to postural alignment and kinesthetic awareness (32, 33). Baduanjin is a specific form of static qigong consisting of eight simple, repetitive movements synchronized with deep breathing (34). It is designed to improve flexibility (35), and regulate the autonomic nervous system (36). Yijinjing is another subtype of qigong that focuses on tendon-strengthening and isometric muscle tension (37). Its movements are performed with deliberate muscular exertion followed by relaxation, aiming to enhance physical strength and vital energy flow (38).

### 2.5 Study selection process

Two authors independently screened the literature in strict accordance with predefined inclusion and exclusion criteria. In cases of discrepancies, the two authors resolved them through discussion; if consensus could not be reached, they consulted a third party for negotiation to reach an agreement. The information extracted from the included studies included the following key details: first author, publication year, country of origin, sample size, sex, age, intervention measures, and outcome indicators.

### 2.6 Quality assessment

**Figure 1:**
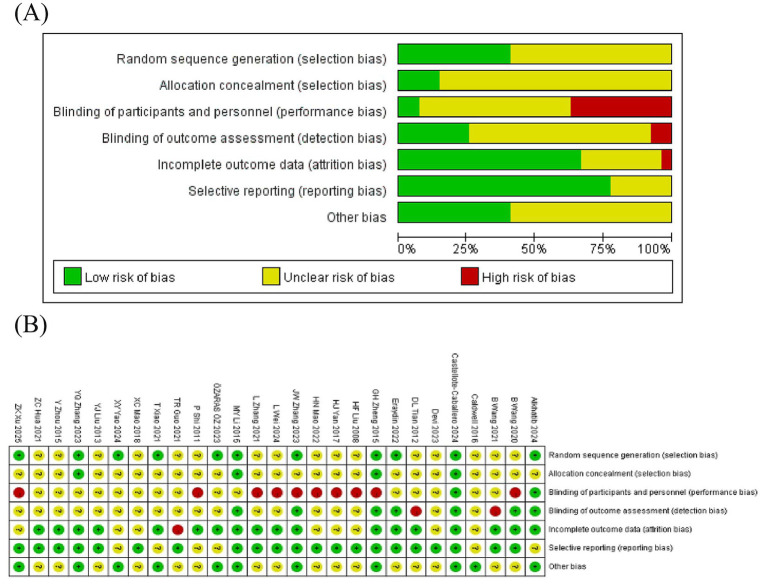
Bias risk assessment of the included studies. **(A)** Risk of bias summary. **(B)** Risk of bias graph.

The quality of all included studies was assessed via the Cochrane risk of bias tool, version 2 (RoB 2.0) (39) and the Grading of Recommendations Assessment, Development, and Evaluation (GRADE) framework. The RoB 2.0 tool evaluates five distinct domains: bias arising from the randomization process, bias due to deviations from intended interventions, bias due to missing outcome data, bias in measurement of the outcomes, and bias in selection of the reported result. Each study was judged as having a “low risk of bias,” “unclear risk of bias,” or a “high risk of bias.” The implementation of blinding was clearly described in all included studies. The high risk of bias was attributed primarily to incomplete outcome data. The results of the risk of bias assessment are summarized in Figure 1. To ensure accuracy, the assessments were independently conducted by two investigators. Any discrepancies were resolved through discussion until a consensus was reached or by consultation with a third reviewer when necessary.

The GRADE framework was applied to rate the overall certainty of evidence for each outcome, incorporating assessments of within-study bias, reporting bias, indirectness, imprecision, heterogeneity, and incoherence. These ratings were ultimately summarized, culminating in a comprehensive evaluation of the quality of evidence.

### 2.7 Statistical analysis

Using R version 4.3.2 (R Foundation for Statistical Computing), a Bayesian NMA was conducted on multiple trial groups employing a prior fuzzy random effects model. Markov chain Monte Carlo methods (40) were employed to obtain optimal pooled estimates and probabilities for each psychomotor exercise type. Model convergence was assessed via trajectory plots and Brooks–Gelman–Rubin plots. Continuous outcomes are expressed as post-erior mean differences (MDs) with 95% credible intervals (CrIs). The Bayesian model was run with 4 chains, each completing 50,000 iterations. The first 20,000 iterations served as burn-in to eliminate initialization effects, with the remainder used for posterior estimation. Model convergence was assessed using trace plots and Gelman–Rubin diagnostics. A *p* value > 0.05 indicated no significant inconsistency between direct and indirect evidence. The surface under the cumulative ranking curve (SUCRA) was calculated to summarize the cumulative probability of each intervention being ranked at each possible position. SUCRA values range from 0 to 100%; a higher SUCRA value indicates a greater probability of being among the most effective interventions. In addition, heterogeneity was quantified using the I^2^ statistic, which describes the percentage of total variation across studies that is due to heterogeneity rather than chance. Network and funnel plots were generated via STATA 15.0 with a loaded pass-through macro. For the network plots, each circle represents a psychomotor type, with edges denoting existing comparisons. The circle size was proportional to the included population size. Cumulative probability plots were created via the ggplot2 package.

## 3 Results

### 3.1 Literature screening results

**Figure 2:**
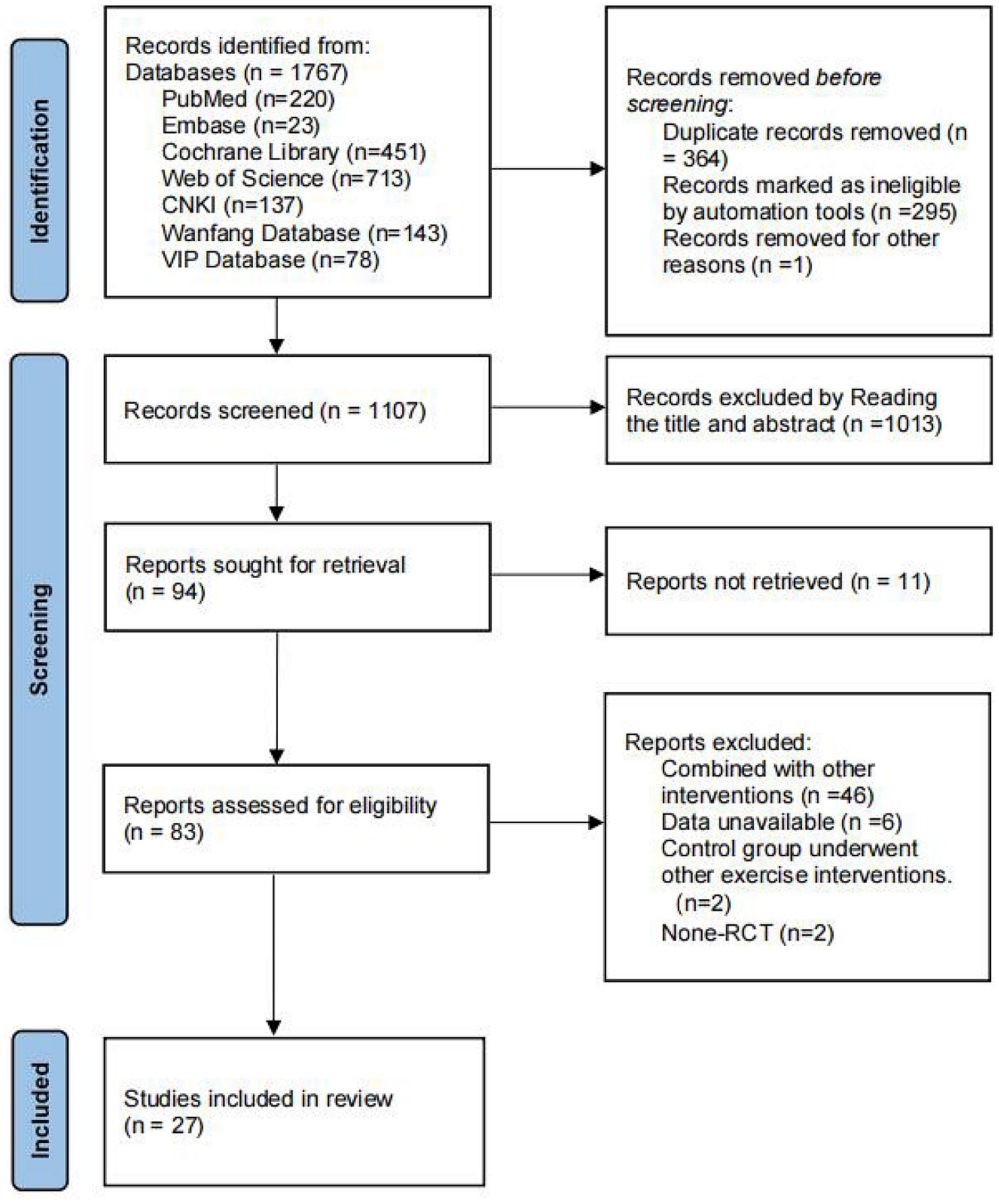
Flow diagram of the literature selection process.

The literature screening process was conducted in strict accordance with the PRISMA flow diagram (41). A total of 1,767 articles were initially identified through the preliminary database search. After preliminary retrieval, 364 duplicates were first excluded. Next, 1,013 articles were excluded on the basis of title and abstract review, and 11 articles were ultimately excluded following full-text reading. Ultimately, 27 articles (6, 36, 37, 42–65) met the final inclusion criteria. The detailed screening process is illustrated in Figure 2.

### 3.2 Basic characteristics of the included studies

Among the 27 studies analyzed, sample sizes ranged from 18 to 206 participants, with the mean age ranging from 18.95 years to 27.87 years. The majority of the studies were carried out in China, and the remaining studies were from India, Turkey, the United States, and Spain.

The included studies evaluated four types of MBTs, with clear correspondence to the study numbers as follows: Baduanjin, Tai Chi; Qigong, yoga, and Yijinjing. Intervention duration and frequency varied across the included studies, and detailed specifics are provided in Table 1. All included studies were RCTs and employed a parallel group design.

**Table 1:** Characteristics of the included studies.

Author/year	Research classification	Country	Sample size *N*	Gender (M/F)	Mean age	Instruments		
						Sleep quality	Anxiety	Depression
Alkhatib et al. (42)	RCT	China	Baduanjin:31 Control:31	0/62	27.87	PSQI		
Liu et al. (49)	RCT	China	Baduanjin:50 Control:50	NR	NR		POMS SCL-90	POMS SCL-90
Liu (37)	RCT	China	Yijinjing:36 Tai chi:36	NR	NR			SDS
Yao et al. (62)	RCT	China	Baduanjin:40 Control:40	9/31	19.23 19.33	PSQI	SRAS	SDS
Yan and Wei (61)	RCT	China	Baduanjin: 50 Control:50	NR	NR		SCL-90	SCL-90
Xiao et al. (59)	RCT	China	Baduanjin:33 Control: 34	24/7 24/10	19.21 19.71		SRAS	
Devi et al. (45)	RCT	India	yoga:20 Control:20	40/0	NR		SCAT	
Özaras (52)	RCT	Turkey	yoga:39 Control:40	8/3 13/27	19.40 19.10		STAI	BDI
Tian (55)	RCT	China	yoga: 30 Control: 30	0/60	NR		SCL-90	SCL-90
Zheng et al. (6)	RCT	China	Tai chi:95 Control: 103	33/62 32/71	20.7 20.6	PSQI		
Zhang (85)	RCT	China	Qigong:30 Control: 30	NR	NR	PSQI	SRAS	SDS
Caldwell et al. (43)	RCT	USA	Tai chi:28 Control: 19	10/18 5/14	21.2 22.4	PSQI	STAI	
Zhang et al. (63)	RCT	China	Tai chi:9 Control: 9	2/7 3/6	24.20 22.50		SRAS	SDS
Mao et al. (50)	RCT	China	Tai chi:52 Control: 52	0/52	NR		SRAS	SDS
Eraydin et al. (46)	RCT	Turkey	yoga:39 Control:40	22/27 18/22	NR		SRAS STAI	
Wang et al. (56)	RCT	China	yoga: 90 Control: 90	NR	NR		SCL-90	SCL90
Hua et al. (48)	RCT	China	yoga: 66 Tai chi: 60 Control: 20	1/66 21/39 10/10	20.29 20.38 21.15		DASS	DASS
Shi et al. (54)	RCT	China	yoga: 38 Control: 38	0/76	NR			POMS
Wei et al. (58)	RCT	China	Baduanjin: 100 Control:100	NR	NR	PSQI		PHQ
Li et al. (36)	RCT	China	Baduanjin: 101 Control:105	15/86 21/84	20.63 20.92	PSQI	POMS	POMS
Mao (50)	RCT	China	Tai chi:17 Control: 16	72/0	20.33		STAI	
Zhou et al. (65)	RCT	China	Qigong A: 25 Qigong B: 25 Control: 25	35/40	NR	PSQI	SRAS	
Castellote-Caballero et al. (44)	RCT	Spain	yoga:65 control:64	33/32 30/34	20.29 20.30		STAI	
Guo (47)	RCT	China	Baduanjin: 30 Control:30	NR	NR		SCL-90	SCL-90
Xu et al. (60)	RCT	China	Tai chi: 60 Control: 60	31/29 32/28	20.6 20.3		SRAS	SDS
Zhang and Jiang (64)	RCT	China	Baduanjin: 34 Control:39	0/73	19.23 19.16		SCL-90	SCL-90
Wang et al. (57)	RCT	China	Baduanjin:100 Control:100	98/102	NR		SCL-90	SCL-90

PSQI, Pittsburgh Sleep Quality Index; POMS, Profile of Mood States; SCL-90, Symptom Checklist-90; HAMD, Hamilton Depression Rating Scale; SDS, Self-Rating Depression Scale; SRAS, Self-Rating Anxiety Scale; HSCL-25, Hopkins Symptom Checklist-25; SCAT, Sport Competition Anxiety Test; BDI, Beck Depression Inventory; STAI, State–Trait Anxiety Inventory; PSS, Perceived Stress Scale; CES-D, Center for Epidemiologic Studies Depression Scale; DASS, Depression Anxiety Stress Scale; SWLS, Satisfaction With Life Scale; PHQ, Patient Health Questionnaire; GAD-7, Generalized Anxiety Disorder-7; MDI, major depression inventor.

### 3.3 Consistency tests

A fundamental premise underlying NMA is the assumption of consistency, which is assessed through consistency tests that examine the concordance between specific bodies of direct and indirect evidence (defined as comparisons) (66). On the basis of the random-effects model adopted for this NMA, both consistency and inconsistency models were fitted to evaluate the agreement between direct and indirect evidence for the three outcome measures. The deviance information criterion (DIC) values and residual deviance for each outcome are presented in Supplementary Table 7. The results from the consistency model for depression, anxiety, and sleep quality yielded model fit statistics of 96.55, 90.45, and 34.47, respectively. The corresponding values from the inconsistency model were 96.37, 90.44, and 34.51. The close similarity between the deviance statistics of the consistency and inconsistency models for all outcomes suggests the model’s outcomes are supported by consistent evidence in most cases. Furthermore, the low I^2^ values of 34, 6, and 4% for depression, anxiety, and sleep quality, respectively, indicate negligible heterogeneity within each network. Given the absence of significant inconsistency and the low heterogeneity, the consistency model was deemed appropriate for estimating comparative effects across all interventions.

### 3.4 Network meta-analysis results

#### 3.4.1 Primary outcome

**Figure 3:**
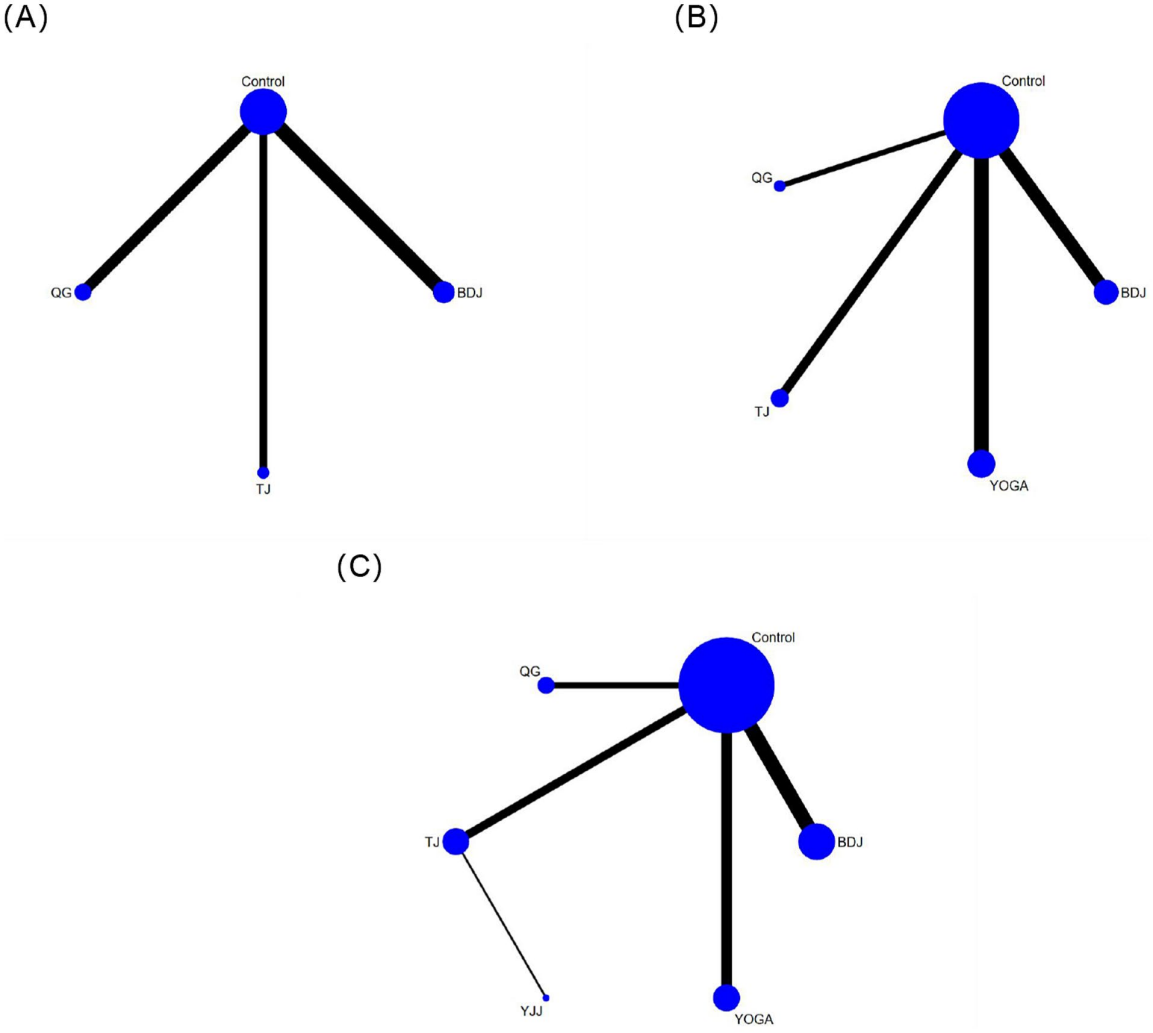
Network meta-analysis intervention diagram. The width of the lines and the size of the nodes are, respectively, proportional to the number of trials comparing each pair of intervention and the number of participants. **(A)** Sleep quality, **(B)** anxiety, **(C)** depression.

The network plot is presented in Figure 3, and the league table summarizing pairwise comparisons is shown in Table 2, pairwise comparisons revealed that the intervention effects of Baduanjin [SMD = −1.29, 95% CrI (−2.67, −0.02)] and Qigong [SMD = −2.24, 95% CrI (−3.85, −0.44)] were significantly better than those of the control group. These findings indicate that, compared with the control group, Baduanjin and Qigong were more effective at promoting sleep quality. Compared with the control group, yoga was associated with a statistically significant improvement in anxiety [SMD = −3.72, 95% CrI (−6.26, −1.47)]. These findings indicate that, compared with the control, yoga is more effective at alleviating anxiety. For depression, the intervention effects of Qigong [SMD = −3.9, 95% CrI (−5.76, −1.37)] were significantly better than those of the control group, and the intervention effects of Qigong were significantly better than those of yoga [SMD = 3.78, 95% CrI (0.96, 5.99)] and Baduanjin [SMD = 3.35, 95% CrI (0.03, 5.27)]. These findings suggest that, compared with the control group, the Qigong group was more effective at alleviating depression, and the Qigong group was more conducive to alleviating depression than the yoga and Baduanjin groups.

**Table 2:** League table of MBTs effects on sleep quality, anxiety and depression.

Sleep quality					
Baduanjin					
−0.57 (−2.87, 1.59)	Tai chi				
0.95 (−1.36, 2.96)	1.53 (−1.04, 3.89)	Qigong			
−1.29 (−2.67, −0.02)*	−0.72 (−2.51, 1.08)	−2.24 (−3.85, −0.44)*	Control		
Anxiety					
Baduanjin					
2.48 (−0.68, 5.92)	yoga				
1.27 (−3.25, 5.72)	−1.23 (−5.93, 3.17)	Qigong			
0.09 (−3.62, 3.87)	−2.39 (−6.27, 1.28)	−1.17 (−5.99, 3.75)	Tai chi		
−1.23 (−3.59, 1.03)	−3.72 (−6.26, −1.47)*	−2.5 (−6.37, 1.40)	−1.32 (−4.34, 1.55)	Control	
Depression					
Baduanjin					
−0.2 (−2.77, 0.74)	yoga				
3.35 (0.03, 5.27)*	3.78 (0.96, 5.99)*	Qigong			
0.08 (−1.18, 2.87)	0.49 (−0.45, 4.12)	−3.05 (−5.21, 1.18)	Tai chi		
−0.45 (−6.66, 5.86)	0.06 (−6.10, 6.77)	−3.65 (−10.04, 3.55)	−0.8 (−6.95, 4.93)	Yijinjing	
−0.41 (−2.14, 0.05)	−0.17 (−1.38, 1.32)	−3.9 (−5.76, −1.37)*	−0.75 (−3.65, 0.03)	−0.19 (−6.68, 5.87)	Control

*p < 0.05.

**Figure 4:**
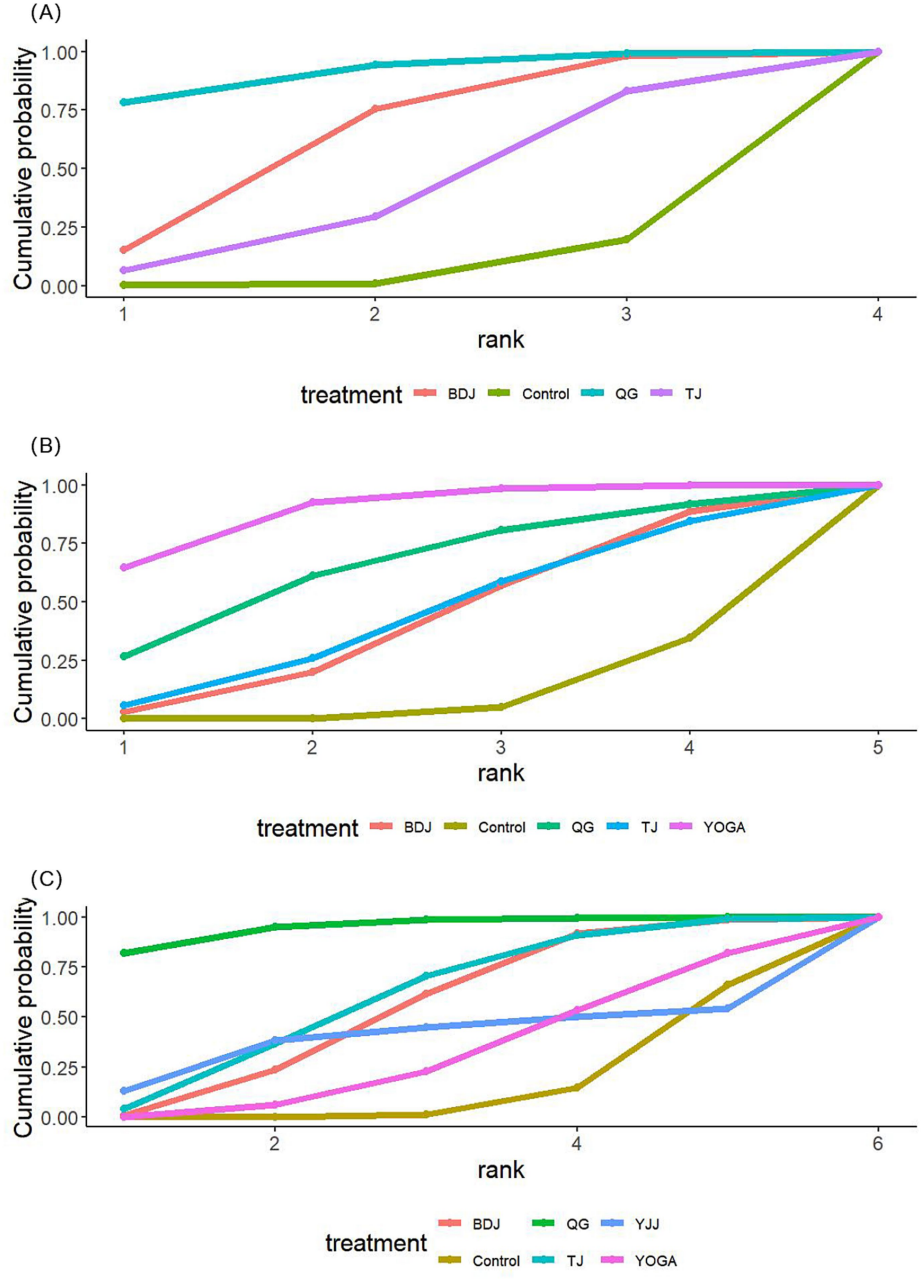
Probability ranking plot of the MBTs effects on various outcomes. **(A)** Sleep quality, **(B)** anxiety, **(C)** depression.

The comparative effectiveness of each MBTs on sleep quality, anxiety, and depression was evaluated on the basis of the SUCRA values in the ranking table (Table 3 and Figure 4). The ranking of the impact of MBTs on sleep quality was as follows: Qigong (SUCRA = 90.53) > Baduanjin (SUCRA = 62.96) > Tai Chi (SUCRA = 39.67). The ranking of the impact of MBTs on anxiety was as follows: yoga (SUCRA = 88.99) > Qigong (SUCRA = 65.10) > Tai Chi (SUCRA= 43.69) > Baduanjin (SUCRA = 42.18). The ranking of the impact of MBTs on depression was as follows: Qigong (SUCRA = 95.04) > Tai Chi (SUCRA = 60.27) > Baduanjin (SUCRA = 55.31) > Yijinjin (SUCRA = 40.07) > yoga (SUCRA = 32.90).

**Table 3:** SUCRA values for the effectiveness of each MBTs.

Treatment	Sleep quality	Anxiety	Depression
Yoga	NR	88.99	32.90
Yijinjing	NR	NR	40.07
Tai chi	39.67	43.69	60.27
Qigong	90.53	65.10	95.04
Control	6.80	10.10	16.43
Baduanjin	62.96	42.18	55.31

#### 3.4.2 Publication bias test

To examine the potential publication bias in the NMA that may be caused by small-scale studies, funnel plots were used for analysis. Funnel plots can be visually inspected for symmetry, which effectively helps us determine whether there is bias caused by small-scale studies, providing an important basis for the reliability of the research results (67). Supplementary Figure 1 shows that the distribution of study points on both sides of the inverted funnel plot is relatively regular, with only a few study points scattered, suggesting that the included literature has a low likelihood of publication bias.

## 4 Discussion

This systematic review and NMA evaluated the effects of various MBTs on sleep quality, anxiety, and depression among college students on 27 RCTs. This study aimed to address a significant gap in previous meta-analyses, which have often been limited by focusing on a single type of MBTs or a single outcome measure, by comprehensively exploring the multidimensional effects of these interventions.

In traditional Chinese medicine and established taxonomies, Baduanjin and Yijinjing are widely recognized as specific subtypes of Qigong. However, in the included randomized controlled trials, these practices were operationalized as distinct, protocolized interventions—each with standardized movement sequences, predefined session frequencies, and independent comparisons against control conditions. Accordingly, to enable direct comparative assessment of their relative effects and to reflect their implementation as discrete programs in real-world university settings, Baduanjin, Yijinjing, and other Qigong forms were modeled as separate nodes in the network.

This configuration does not contradict the traditional classification of these practices within the Qigong family. Rather, it is a methodological necessity for evaluating the comparative effectiveness of specific, commonly studied intervention protocols. In this framework, a high ranking of the aggregated ‘Qigong’ node suggests a broad, shared beneficial effect across this category of mindful movement interventions, whereas the independent performance of Baduanjin or Yijinjing provides protocol-specific evidence for their potential application.

### 4.1 Effects of MBTs on sleep quality

On the basis of the results of this NMA, Qigong was identified as the most effective MBTs for improving sleep quality. Previous meta-analyses (68–70) have endorsed MBTs for sleep quality. However, robust evidence supporting the superiority of individual MBTs is lacking, as the majority of studies comparing MBTs as a group with other exercise types. For example, both traditional Chinese exercise (a typical category of MBTs) and aerobic exercise significantly improve sleep quality among college students, and quantitative evidence from meta-analyses indicates that aerobic exercise is more effective than MBTs are (71, 72). Additionally, systematic comparisons across different categories of MBTs demonstrated that, compared with Tai Chi and general aerobic exercise, Qigong induces greater improvements in sleep outcomes among older generations (73). The superior efficacy of Qigong may be attributed to its integrative nature, which potentially combines the advantages of various traditional Chinese exercises (74). Qigong primarily encompasses two forms (75): static exercises (e.g., Baduanjin, Liuzijue) and dynamic exercises (e.g., Tai Chi, Wuqinxi, Yijinjing). Both forms have positive effects on disease prevention, treatment, and rehabilitation and promote physical and mental wellbeing (70). On the other hand, multicomponent traditional Chinese exercises can positively impact multiple core dimensions of sleep quality, including subjective sleep quality, sleep latency, sleep duration, habitual sleep efficiency, sleep disturbances, the use of hypnotic medication, and daytime dysfunction (72). Consequently, Qigong has enhanced the improvement of sleep quality.

### 4.2 Effects of MBTs on anxiety symptoms

This NMA identified yoga as the most effective MBTs for alleviating anxiety symptoms among college students. While previous meta-analyses have evaluated the clinical efficacy of individual interventions—such as yoga (76), Tai Chi (15, 77), Baduanjin (16), and Qigong (78)—and have improved our understanding of each modality’s benefits, they have generally been limited to single-therapy evaluations and lack direct comparisons across different MBTs. This phenomenon may be attributed to the modulatory impact of yoga on anxiety, as evidenced by studies indicating that its superior efficacy may stem from its unique ability to address the somatic and cognitive facets of anxiety concurrently. For example, while tai chi also promotes relaxation, its learning curve might be steeper. In contrast, the more immediate physical engagement and explicit focus on breathwork in yoga practices (e.g., laughter yoga) can induce rapid downregulation of the stress response (e.g., reducing salivary cortisol) (20) and alter emotional processing (79). These effects may be particularly potent for the rapid relief of anxiety symptoms in young adults. In addition, yoga has been found to reduce the levels of proinflammatory cytokines, including TNF-*α* and IL-1β (80), thereby improving anxiety symptoms via the modulation of neuroinflammatory pathways—a finding that is consistent with established neuroimmune mechanisms of stress-related anxiety. The pathways discussed above are drawn from separate investigations and are offered here as theoretical context, not as conclusions supported by the present data. Future research incorporating mechanistic measures is needed to test these hypotheses directly. Through these combined pathways, yoga facilitates rapid relaxation and helps mitigate academic stress, representing a practical and readily implementable intervention in university settings. Furthermore, a critical evidence gap exists regarding their comparative efficacy. Our NMA directly addresses this gap by simultaneously ranking multiple MBTs, thereby identifying yoga as the most promising intervention for anxiety among college students.

### 4.3 Effects of MBTs on depressive symptoms

The study concluded that Qigong is the most effective intervention for improving depressive symptoms among college students. Extensive research has demonstrated that Qigong exercises significantly reduce anxiety and depression symptoms (29, 81). However, previous studies have focused primarily on evaluating single-modality interventions, and a direct comparative assessment of the relative efficacy across different MBTs is lacking. The underlying mechanism for the antidepressant effect of Qigong may be its impact on central nervous system excitation. Specifically, qigong training increases excitation in the middle cerebral cortex, which subsequently modulates several key neurobiological pathways. These include the hypothalamic–pituitary–adrenal (HPA) axis, monoamine neurotransmitter systems, brain-derived neurotrophic factor (BDNF) expression, and adiponectin levels (82–84). The modulation of these particular pathways is critically aligned with the contemporary neurobiological understanding of depression, offering a plausible theoretical explanation for the broad effects of Qigong observed in clinical studies. However, it is important to emphasize that none of the trials included in this meta-analysis measured these biomarkers. The proposed mechanisms are therefore speculative and are presented solely to contextualize the observed efficacy within a broader theoretical framework. This concordance may explain why the broad, systemic action of Qigong demonstrates superior efficacy for depression than other MBTs, which might target more specific mechanisms. Therefore, Qigong represents a comprehensive nonpharmacological intervention are more effective in promoting sleep quality among college students.

### 4.4 Clinical implications

Currently, few systematic comparisons exist regarding the effects of various MBTs on anxiety, depression, and sleep quality among college students. Furthermore, the lack of standardized intervention protocols in practical applications may, to some extent, limit the comprehensiveness and accuracy of existing findings. Therefore, future research should prioritize comparative investigations conducted within a unified framework to comprehensively evaluate the collective impact of different MBTs on depression, anxiety, and sleep quality. Such efforts will enable a scientific assessment of the efficacy of MBTs in alleviating these symptoms and help establish optimal, symptom-specific application strategies for different MBTs types.

### 4.5 Strengths and limitations

The main strength of this study is the apparent symptom specificity of MBTs among college students, whereas yoga was ranked first for alleviating anxiety and Qigong for improving sleep quality. Qigong emerged as the most effective intervention for depressive symptoms. This tripartite outcome suggests that the interrelated mechanisms underpinning sleep, anxiety, and depression may be differentially modulated by distinct MBTs. The multifaceted physiological impact of Qigong appears to be particularly well suited to counter the neurobiological dysfunctions characteristic of depressive states. Furthermore, the literature search and study selection processes were rigorously conducted in accordance with current guidelines and included multiple electronic databases to ensure comprehensive coverage. Our NMA included only RCTs, which represent the optimal study design for investigating causal relationships. The findings of this research are anticipated to provide valuable evidence to support informed decision-making by healthcare professionals, educators, and policy makers. Subsequent studies should prioritize the development of tailored intervention strategies on the basis of individual characteristics, including gender and health status, as well as specific implementation contexts, to increase the effectiveness of approaches aimed at improving various negative emotional states and sleep quality.

Several limitations exist for this NMA: ① the inclusion of only Chinese and English publications may inevitably lead to some omissions. ② The majority of included trials have certain unclear risks in their RCTs, mainly concentrated in areas including random sequence generation, blinding of outcome assessment, allocation concealment (selection bias), blinding of participants and personnel (performance bias), and blinding of outcome assessment (detection bias). This may affect the generalizability and reliability of the study conclusions. ③ Most included studies did not provide data on whether participants continued to practice MBTs after the intervention period. Owing to the lack of follow-up data, the long-term effects of MBTs on college students’ anxiety, depression, and sleep quality remain unclear.

## 5 Conclusion

In conclusion, this study systematically integrates 27 RCTs and, through a NMA approach, compares the effectiveness of mainstream MBTs on anxiety, depression, and sleep quality. With a focus on the perspective of MBTs types and their intervention effects on different outcome indicators, the findings indicate that Qigong has the optimal intervention effect in alleviating depression and improving sleep quality, whereas yoga has the greatest effectiveness in alleviating anxiety. This finding addresses the limitations of previous studies that often focused on single-modality interventions or solitary health outcomes. Through a comprehensive analysis of the effects of different MBTs across multiple outcome measures, our study provides valuable insights for selecting optimal intervention strategies tailored to specific symptoms. However, owing to limitations in the number and quality of included studies, as well as potential selection bias, caution should be exercised when interpreting the results of this study. Therefore, future research should further expand the sample size, optimize the study design, and conduct more studies on the optimal dose of each therapy. Notably, in the selection of control groups, active controls should be adopted to effectively mitigate the interference of placebo effects, thereby enhancing the reliability of research findings.

## Data Availability

The datasets presented in this study can be found in online repositories. The names of the repository/repositories and accession number(s) can be found in the article/Supplementary material.

## References

[1] Guerriero MA Dipace A Monda A De Maria A Polito R Messina G . Relationship between sedentary lifestyle, physical activity and stress in university students and their life habits: a scoping review with PRISMA checklist (PRISMA-ScR). Brain Sci. (2025) 15:78. doi: 10.3390/brainsci15010078, 39851445 PMC11763463

[2] Ye L Wang B Qiu F. Effects of physical activity on mental and behavioral health and functioning for college students: a systematic review using ICF. Chin. J. Rehab. Theor. Prac. (2023) 22:38–47. doi: 10.3969/j.issn.1006-9771.2023.01.006

[3] Auerbach RP Mortier P Bruffaerts R Alonso J Benjet C Cuijpers P . WHO world mental health surveys international college student project: prevalence and distribution of mental disorders. J Abnorm Psychol. (2018) 127:623–38. doi: 10.1037/abn0000362 30211576 PMC6193834

[4] Tosevski DL Milovancevic MP Gajic SD. Personality and psychopathology of university students. Curr Opin Psychiatry. (2010) 23:48–52. doi: 10.1097/YCO.0b013e328333d625, 19890212

[5] García-Pérez L Atencia-Rodriguez ME Cepero-González M Padial-Ruz R. Effectiveness of physical activity, mindfulness and mind-body therapies in improving mental health of university students: a systematic review of RCTS. J Am Coll Heal. (2025) 11:1–16. doi: 10.1080/07448481.2025.2492174 40262195

[6] Zheng G Lan X Li M Ling K Lin H Chen L . Effectiveness of tai chi on physical and psychological health of college students: results of a randomized controlled trial [journal article]. PLoS One. (2015) 10:e0132605. doi: 10.1371/journal.pone.0132605, 26147842 PMC4492604

[7] Dyrbye LN Thomas MR Shanafelt TD. Systematic review of depression, anxiety, and other indicators of psychological distress among US and Canadian medical students. Acad Med. (2006) 81:9. doi: 10.1097/00001888-200604000-00009 16565188

[8] Li J Zhou Z Hao S Zang L. Optimal intensity and dose of exercise to improve university students’ mental health: a systematic review and network meta-analysis of 48 randomized controlled trials. Eur J Appl Physiol. (2025) 125:1395–410. doi: 10.1007/s00421-024-05688-9, 39692765

[9] Powell KE King AC Buchner DM Campbell WW DiPietro L Erickson KI . The scientific foundation for the physical activity guidelines for Americans, 2nd edition. J Phys Act Health. (2018) 22:1–11. doi: 10.1123/jpah.2018-0618 30558473

[10] Strehli I Burns RD Bai Y Ziegenfuss DH Block ME Brusseau TA. Mind-body physical activity interventions and stress-related physiological markers in educational settings: a systematic review and Meta-analysis. Int J Environ Res Public Health. (2020) 18:224. doi: 10.3390/ijerph18010224, 33396730 PMC7795448

[11] Winbush NY Gross CR Kreitzer MJ. The effects of mindfulness-based stress reduction on sleep disturbance: a systematic review. Explore. (2007) 3:585–91. doi: 10.1016/j.explore.2007.08.003, 18005910

[12] Russo MA Santarelli DM O'Rourke D. The physiological effects of slow breathing in the healthy human. Breathe (Sheff). (2017) 13:298–309. doi: 10.1183/20734735.009817, 29209423 PMC5709795

[13] Hölzel BK Carmody J Vangel M Congleton C Yerramsetti SM Gard T . Mindfulness practice leads to increases in regional brain gray matter density. Psychiatry Res Neuroimaging. (2011) 191:36–43. doi: 10.1016/j.pscychresns.2010.08.006, 21071182 PMC3004979

[14] Cui J Lin J Liu Y Wang P Zeng H. Mental health benefits of tai chi for college students: a systematic review using ICF. Chin J Rehabil Theory Pract. (2023) 22:48–54. doi: 10.3969/j.issn.1006-9771.2023.01.007

[15] Wang G Liu Y Peng C Shen T Du B Yi L. Physical and psychological impacts of tai chi on college students and the determination of optimal dose: a systematic review and meta-analysis of randomized controlled trials. Eur J Integr Med. (2025) 76:102450. doi: 10.1016/j.eujim.2025.102450

[16] Cheng FK. Effects of Baduanjin on mental health: a comprehensive review. J Bodyw Mov Ther. (2015) 19:138–49. doi: 10.1016/j.jbmt.2014.11.001, 25603754

[17] Bushnell GA Gerhard T Keyes K Hasin D Cerda M Olfson M. Association of Benzodiazepine Treatment for sleep disorders with drug overdose risk among young people. JAMA Netw Open. (2022) 5:e2243215. doi: 10.1001/jamanetworkopen.2022.43215, 36413369 PMC9682430

[18] Dubreucq J Plasse J Franck N. Self-stigma in serious mental illness: a systematic review of frequency, correlates, and consequences. Schizophr Bull. (2021) 47:1261–87. doi: 10.1093/schbul/sbaa181, 33459793 PMC8563656

[19] Moitra M Santomauro D Collins PY Vos T Whiteford H Saxena S . The global gap in treatment coverage for major depressive disorder in 84 countries from 2000-2019: a systematic review and Bayesian meta-regression analysis. PLoS Med. (2022) 19:e1003901. doi: 10.1371/journal.pmed.1003901, 35167593 PMC8846511

[20] Erkin O Kocacal E. The impact of laughter yoga as a NIC on health parameters in nurses and nursing students: a systematic review. BMC Complement Med Ther. (2024) 24:378. doi: 10.1186/s12906-024-04663-3, 39472871 PMC11520822

[21] Bennett-Weston A Keshtkar L Jones M Sanders C Lewis C Nockels K . Interventions to promote medical student wellbeing: an overview of systematic reviews. BMJ Open. (2024) 14:e082910. doi: 10.1136/bmjopen-2023-082910, 38724055 PMC11086405

[22] Chimiklis AL Dahl V Spears AP Goss K Fogarty K Chacko A. Yoga, mindfulness, and meditation interventions for youth with ADHD: systematic review and meta-analysis. J Child Fam Stud. (2018) 27:3155–68. doi: 10.1007/s10826-018-1148-7

[23] Gibbons J. Does yoga improve mental health in adolescence? A systematic review exploring the impact of yoga interventions implemented within secondary education. Cham: Springer (2022).

[24] Palacios AM Bender SL Berry DJ. Characteristics of mindfulness-based interventions in schools: a systematic review in school psychology journals. Contemp Sch Psychol. (2023) 27:182–97. doi: 10.1007/s40688-022-00432-6, 36274976 PMC9575630

[25] Kodama S Fujihara K Horikawa C Yamada M Sato T Yaguchi Y . Network Meta-analysis of drug therapies for lowering uric acid and mortality risk in patients with heart failure. Cardiovasc Drugs Ther. (2021) 35:1217–25. doi: 10.1007/s10557-020-07097-4, 33095357

[26] Tonin FS Rotta I Mendes AM Pontarolo R. Network meta-analysis: a technique to gather evidence from direct and indirect comparisons. Pharm Pract. (2017) 15:943. doi: 10.18549/PharmPract.2017.01.943, 28503228 PMC5386629

[27] Chandler J Cumpston M Li T Page MJ Welch V. Cochrane handbook for systematic reviews of interventions. Hoboken: Wiley (2019). 10.1002/14651858.ED000142 PMC10284251 31643080

[28] Moher D Shamseer L Clarke M Ghersi D Liberati A Petticrew M. Preferred reporting items for systematic review and meta-analysis protocols (PRISMA-P) 2015 statement. Syst Rev. (2015) 4:1. doi: 10.1186/2046-4053-4-1 25554246 PMC4320440

[29] Lin J Gao YF Guo Y Li M Zhu Y You R . Effects of qigong exercise on the physical and mental health of college students: a systematic review and Meta-analysis. BMC Complement Med Ther. (2022) 22:287. doi: 10.1186/s12906-022-03760-5, 36348349 PMC9641907

[30] Yeh ML Chung YC. A randomized controlled trial of qigong on fatigue and sleep quality for non-Hodgkin’s lymphoma patients undergoing chemotherapy. Eur J Oncol Nurs. (2016) 23:81–6. doi: 10.1016/j.ejon.2016.05.003, 27456379

[31] Cramer H Lauche R Anheyer D Pilkington K de Manincor M Dobos G . Yoga for anxiety: a systematic review and meta-analysis of randomized controlled trials. Depress Anxiety. (2018) 35:830–43. doi: 10.1002/da.22762, 29697885

[32] Li F Harmer P Fitzgerald K Eckstrom E Stock R Galver J . Tai chi and postural stability in patients with Parkinson's disease. N Engl J Med. (2012) 366:511–9. doi: 10.1056/NEJMoa1107911, 22316445 PMC3285459

[33] Wayne PM Kaptchuk TJ. Challenges inherent to t'ai chi research: part I—t'ai chi as a complex multicomponent intervention. J Altern Complement Med. (2008) 14:95–102. doi: 10.1089/acm.2007.7170A, 18199021

[34] Zou L Sasaki JE Wang H Xiao Z Fang Q Zhang M. A systematic review and meta-analysis of Baduanjin qigong for health benefits: randomized controlled trials. Evid Based Complement Alternat Med. (2017) 2017:4548706. doi: 10.1155/2017/4548706, 28367223 PMC5359459

[35] Zou L Yeung A Quan X Boyden SD Wang H. A systematic review and meta-analysis of mindfulness-based (Baduanjin) exercise for alleviating musculoskeletal pain and improving sleep quality in people with chronic diseases. Int J Environ Res Public Health. (2018) 15:206. doi: 10.3390/ijerph15020206, 29370149 PMC5858275

[36] Li M Fang Q Li J Zheng X Tao J Yan X . The effect of Chinese traditional exercise-Baduanjin on physical and psychological well-being of college students: a randomized controlled trial. PLoS One. (2015) 10:e0130544. doi: 10.1371/journal.pone.0130544, 26158769 PMC4497728

[37] Liu. The study Yijinjing and taijiquan influence of depression on university students. Sichuan Sports Science. (2013) 32:49–51. doi: 10.13932/j.cnki.sctykx.2013.06.019.

[38] Zhang M Xv G Luo C Meng D Ji Y. Qigong Yi Jinjing promotes pulmonary function, physical activity, quality of life and emotion regulation self-efficacy in patients with chronic obstructive pulmonary disease: a pilot study. J Altern Complement Med. (2016) 22:810–7. doi: 10.1089/acm.2015.0224 27487437

[39] Sterne JA Page MJ Elbers RG Blencowe NS Boutron I. Rob 2: A revised tool for assessing risk of bias in randomised trials. Cham: Springer (2019). 10.1136/bmj.l4898 31462531

[40] Jansen JP Crawford B Bergman G Stam W. Bayesian meta-analysis of multiple treatment comparisons: an introduction to mixed treatment comparisons. Value Health. (2008) 11:956–64. doi: 10.1111/j.1524-4733.2008.00347.x, 18489499

[41] Page MJ McKenzie JE Bossuyt PM Boutron I Hoffmann TC Mulrow CD . The PRISMA 2020 statement: an updated guideline for reporting systematic reviews. BMJ. (2021) 372:n71. doi: 10.1136/bmj.n71 33782057 PMC8005924

[42] Alkhatib A Ahmad HA Zhang C Peng W Li X. Impact of traditional Chinese Baduanjin exercise on menstrual health among international female students studying in China: a randomized controlled trial. Front Public Health. (2024) 12:1259634. doi: 10.3389/fpubh.2024.1259634, 38384881 PMC10879288

[43] Caldwell KL Bergman SM Collier SR Triplett NT Quin R Bergquist J . Effects of tai chi chuan on anxiety and sleep quality in young adults: lessons from a randomized controlled feasibility study. Nat Sci Sleep. (2016) 8:305–14. doi: 10.2147/nss.S117392 27895522 PMC5118018

[44] Castellote-Caballero Y Carcelen-Fraile M d C Aibar-Almazan A Rivas-Campo Y Gonzalez-Martin AM. Yoga as a therapeutic approach to mental health in university students: a randomized controlled trial. Front Public Health. (2024) 12:1406937. doi: 10.3389/fpubh.2024.1406937, 38903593 PMC11188441

[45] Devi KS Singh MN. Effect of yogic practice on mental toughness and anxiety of physical education students. Afr J Biol Sci. (2024) 6:5860–7. doi: 10.48047/AFJBS.6.Si4.2024.5860-5867

[46] Eraydin C Alpar SE. The effect of laughter therapy on nursing students' anxiety, satisfaction with life, and psychological well-being during the COVID-19 pandemic: randomized controlled study. Adv. Int. Med. (2022) 9:173–9. doi: 10.1016/j.aimed.2022.06.006, 35782290 PMC9232266

[47] Guo T. An experimental study on the effects of the eight brocades on college students' mental health. Stu. Sci. Eng. RTVU. (2021) 2:76–8. doi: 10.19469/j.cnki.1003-3297.2021.02.0076

[48] Hua Z Sun J. Effects of mind-body practice on anxiety, depression and stress of college students. J. Guangzhou Sport Univ. (2021) 41:95–102. doi: 10.13830/j.cnki.cn44-1129/g8.2021.01.022

[49] Liu H An H Wang C Cao C Zhang B. Psychological effects of health qigong Ba duan Jin. J. Wuhan Insti. Phys. Educ. (2008) 1:54–57+77. doi: 10.15930/j.cnki.wtxb.2008.01.021

[50] Mao X. The effect of three different types of exercise intervention methods on college students with their state and trait anxiety. J. Jilin Sport Univ. (2018) 34:53–8. doi: 10.13720/j.cnki.22-1286.2018.05.010

[51] Mao H Yuan L Luo M. The effect of tai chi on depression and anxiety levels among female college students. New West. (2008) 4:199–232. doi: 10.3969/j.issn.1009-8607-B.2008.04.162

[52] Özaras G. Effect of laughter yoga on anxiety, depression and physiological parameters in nursing students during the COVID-19 pandemic: a randomized controlled study. Turk Klin J Nurs Sci. (2023) 15:1104–13. doi: 10.5336/nurses.2023-98713

[53] Shan Q Wei B Wei Z. Effects of yoga on depression among college students. J Beijing Sport Univ. (2007) S1:140–53. doi: 10.19582/j.cnki.11-3785/g8.2007.s1.072

[54] Shi P Huang M. Effects of yoga exercise on female college students' mood and serum ACTH levels. Sports. (2011) 13:71–3. doi: 10.3969/j.issn.1674-151x.2011.11.034

[55] Tian D. Effects of yoga exercise prescription on physiological function of female students of higher vocational school. J. Xichang College·Nat. Sci.Edition. (2012) 26:139–41. doi: 10.3969/j.issn.1673-1891.2012.02.043

[56] Wang B Peng Y Mao Y. Research on the impact of online yoga on the mental health of college students under the new crown pneumonia epidemic. Psychol. (2020) 15:87–9. doi: 10.19738/j.cnki.psy.2020.19.034

[57] Wang B Wang X Chang Y Mao Y Peng Y. Research on the effect of online practice of the eight brocades on the mental health of university freshmen during the COVID-19 pandemic. Health Voc. Edu. (2021) 39:155–9.

[58] Wei L Min R Zhang J Cai Y. Experiment research on regulating the depression and sleep of college students with Baduanjin exercise. Contemporary Sports Technol. (2024) 14:104–106+111. doi: 10.16655/j.cnki.2095-2813.2024.17.029

[59] Xiao T Jiao C Yao J Yang L Zhang Y Liu S . Effects of basketball and Baduanjin exercise interventions on problematic smartphone use and mental health among college students: a randomized controlled trial. Evid Based Complement Alternat Med. (2021) 2021:8880716. doi: 10.1155/2021/8880716, 33574886 PMC7864751

[60] Xu Z Yao L Guo Q Liu Z Wu J Wang S . The impact of tai chi combined with music-assisted intervention on depression and anxiety among college students. Basic Appl Soc Psychol. (2025) 47:85. doi: 10.1080/01973533.2025.2494085

[61] Yan H Wei Q. Research on the effects of the eight brocades on the psychological state and physical fitness regulation of college students. Sports. (2017) 23:49–50. doi: 10.3969/j.issn.1674-151x.2017.23.024

[62] Yao X Xiong L Ouyang Y Wang H Zhu L. Research on the intervention effect of five-element music combined with eight-section brocade on depression among medical students in higher vocational colleges. Front Psychol. (2024) 15:1439713. doi: 10.3389/fpsyg.2024.1439713, 39403247 PMC11471634

[63] Zhang J Gao T Li Y Song Z Cui M Wei Q . The effect of Bafa Wubu of tai chi on college students' anxiety and depression: a randomized, controlled pilot study. Front Physiol. (2023) 14:1036010. doi: 10.3389/fphys.2023.1036010, 36760533 PMC9905723

[64] Zhang Y Jiang X. The effect of Baduanjin exercise on the physical and mental health of college students. Medicine. (2023) 102:e34897. doi: 10.1097/md.0000000000034897 37653828 PMC10470797

[65] Zhao Y Zhao M Zhao S Liang L. On the influences of the gymnastic qigong and 12 style Daoyin Yangsheng gong to the undergraduates’sleeping quality and emotion. Liaoning Sport Sci. Tecnol. (2015) 37:52–4. doi: 10.13940/j.cnki.lntykj.2015.04.018

[66] Dias S Welton NJ Sutton AJ Caldwell DM Lu G Ades A. Evidence synthesis for decision making 4: inconsistency in networks of evidence based on randomized controlled trials. Med Decis Mak. (2013) 33:641–56. doi: 10.1177/0272989X12455847, 23804508 PMC3704208

[67] Khera R Murad MH Chandar AK Dulai PS Wang Z Prokop LJ . Association of Pharmacological Treatments for obesity with weight loss and adverse events: a systematic review and Meta-analysis. JAMA. (2016) 315:2424–34. doi: 10.1001/jama.2016.7602, 27299618 PMC5617638

[68] Chan JS Ho RT Chung KF Wang CW Yao TJ Ng SM . Qigong exercise alleviates fatigue, anxiety, and depressive symptoms, improves sleep quality, and shortens sleep latency in persons with chronic fatigue syndrome-like illness. Evid Based Complement Alternat Med. (2014) 2014:106048. doi: 10.1155/2014/106048, 25610473 PMC4290154

[69] Kong H Zhao Y Yuan Q Li Z Wang F. Meta analysis of the intervention effect of physical exercise on sleep quality and state of mind of Chinese college students. Sports Res. Educ. (2019) 34:85–91. doi: 10.16207/j.cnki.2095-235x.2019.05.016

[70] Wu WW Kwong E Lan XY Jiang XY. The effect of a meditative movement intervention on quality of sleep in the elderly: a systematic review and Meta-analysis. J Altern Complement Med. (2015) 21:509–19. doi: 10.1089/acm.2014.0251, 26120865

[71] Li L Wang C Wang D Li H Zhang S He Y . Optimal exercise dose and type for improving sleep quality: a systematic review and network meta-analysis of RCTs. Front Psychol. (2024) 15:1466277. doi: 10.3389/fpsyg.2024.1466277, 39421847 PMC11484100

[72] Yang Z Zhai H Yang Z Ning B. Comparing the efficacy of traditional Chinese exercises and general aerobic exercises in university students with sleep disorders: a systematic review and meta-analysis. Medicine. (2024) 103:e38521. doi: 10.1097/MD.0000000000038521, 38847687 PMC11155542

[73] He WB Gao YY Han XM. Effects of traditional Chinese exercises and general aerobic exercises on older adults with sleep disorders: a systematic review and meta-analysis. J Integr Med. (2021) 19:7. doi: 10.1016/j.joim.2021.09.007, 34649821

[74] Liu F Cui J Liu X Chen KW Chen X Li R. The effect of tai chi and qigong exercise on depression and anxiety of individuals with substance use disorders: a systematic review and meta-analysis. BMC Complement Med Ther. (2020) 20:161. doi: 10.1186/s12906-020-02967-8, 32471415 PMC7260819

[75] Wang X Li P Pan C Dai L Wu Y Deng Y. The effect of mind-body therapies on insomnia: a systematic review and Meta-analysis. Evid Based Complement Alternat Med. (2019) 2019:9359807. doi: 10.1155/2019/9359807, 30894878 PMC6393899

[76] Lin Y Gao W. The effects of physical exercise on anxiety symptoms of college students: a meta-analysis. Front Psychol. (2023) 14:1136900. doi: 10.3389/fpsyg.2023.1136900, 37063553 PMC10100500

[77] Litwiller F White C Hamilton-Hinch B Gilbert R. The impacts of recreation programs on the mental health of postsecondary students in North America: an integrative review. Leis Sci. (2018) 44:96–120. doi: 10.1080/01490400.2018.1483851

[78] Xu Y. Research progress on the application of shaolin YiJinJing in public mental health. Wushu Stud. (2022) 7:85–96. doi: 10.13293/j.cnki.wskx.009358

[79] Herbert C. Enhancing mental health, well-being and active lifestyles of university students by means of physical activity and exercise research programs. Front Public Health. (2022) 10:849093. doi: 10.3389/fpubh.2022.849093, 35548074 PMC9082407

[80] Lurie DI. An integrative approach to neuroinflammation in psychiatric disorders and neuropathic pain. J Exp Neurosci. (2018) 12:1179069518793639. doi: 10.1177/1179069518793639, 30127639 PMC6090491

[81] Liu X Clark J Siskind D Williams GM Byrne G Yang JL . A systematic review and meta-analysis of the effects of qigong and tai chi for depressive symptoms. Complement Ther Med. (2015) 23:516–34. doi: 10.1016/j.ctim.2015.05.001, 26275645

[82] Chan JS Li A Ng SM Ho RT Xu A Yao TJ . Adiponectin potentially contributes to the antidepressive effects of Baduanjin qigong exercise in women with chronic fatigue syndrome-like illness. Cell Transplant. (2017) 26:493–501. doi: 10.3727/096368916X694238, 27938498 PMC5657703

[83] Tsang HWH Fung KK. A review on neurobiological and psychological mechanisms underlying the anti-depressive effect of qigong exercise. J Health Psychol. (2008) 13:857–63. doi: 10.1177/1359105308095057, 18809635

[84] Tsang HWH Tsang WWN Jones AYM Fung KMT Chan AHL Chan EP . Psycho-physical and neurophysiological effects of qigong on depressed elders with chronic illness. Aging Ment Health. (2013) 17:336–48. doi: 10.1080/13607863.2012.732035, 23072658

[85] Zhang L. Influence of Traditional Physical Fitness on Psychological State of College Students during COVID-19 Epidemic Period. Journal of Yanan University (Natural Science Edition), (2021) 40:94–98. doi: 10.13876/J.cnki.ydnse.2021.01.094, 23072658

