# Supplementary material for: Effect of mind–body therapies on anxiety, depression and sleep quality in college students: a network meta-analysis
Source: Front Public Health. 2026 Mar 19;14:1767300. doi: 10.3389/fpubh.2026.1767300 (PMC13043389; doi:10.3389/fpubh.2026.1767300)
Supplement: Supplementary file 1 [file Table_1.docx]

Supplementary Material

# Supplementary Tables

| **Steps** | Search Strategies |
| --- | --- |
| **#1** | MeSH descriptor: [Students] explode all trees |
| **#2** | (Students):ti,ab,kw OR (Student):ti,ab,kw OR (School Enrollment):ti,ab,kw OR (Enrollment, School):ti,ab,kw OR (Enrollments, School):ti,ab,kw |
| **#3** | (School Enrollments):ti,ab,kw OR (College students):ti,ab,kw OR (High school students):ti,ab,kw OR (Middle school student):ti,ab,kw |
| **#4** | #1 or #2 or #3 |
| **#5** | MeSH descriptor: [Anxiety] explode all trees |
| **#6** | MeSH descriptor: [Depression] explode all trees |
| **#7** | MeSH descriptor: [Sleep] explode all trees |
| **#8** | (Anxiety):ti,ab,kw OR (Angst):ti,ab,kw OR (Nervousness):ti,ab,kw OR (Hypervigilance):ti,ab,kw OR (Social Anxiety):ti,ab,kw |
| **#9** | (Anxieties, Social):ti,ab,kw OR (Anxiety, Social):ti,ab,kw OR (Social Anxieties):ti,ab,kw OR (Anxiousness):ti,ab,kw OR (Depression):ti,ab,kw |
| **#10** | (Depressive Symptoms):ti,ab,kw OR (Depressive Symptom):ti,ab,kw OR (Symptom, Depressive):ti,ab,kw OR (Emotional Depression):ti,ab,kw OR (Depression, Emotional):ti,ab,kw |
| **#11** | (Sleep):ti,ab,kw OR (Sleeping Habits):ti,ab,kw OR (Sleep Habits):ti,ab,kw OR (Habit, Sleep):ti,ab,kw OR (Habits, Sleep):ti,ab,kw |
| **#12** | (Sleep Habit):ti,ab,kw OR (Sleeping Habit):ti,ab,kw OR (Habit, Sleeping):ti,ab,kw OR (Habits, Sleeping):ti,ab,kw |
| **#13** | #5 or #6 or #7 or #8 or #9 or #10 or #11 or #12 |
| **#14** | MeSH descriptor: [Mind-Body Therapies] explode all trees |
| **#15** | (Mind-Body Therapies):ti,ab,kw OR (Mind Body Therapies):ti,ab,kw OR (Mind-Body Therapy):ti,ab,kw OR (Therapies, Mind-Body):ti,ab,kw OR (Therapy, Mind-Body):ti,ab,kw |
| **#16** | (Mind-Body Medicine):ti,ab,kw OR (Mind Body Medicine):ti,ab,kw OR (Tai Ji):ti,ab,kw OR (Tai-ji):ti,ab,kw OR (Tai Chi):ti,ab,kw |
| **#17** | (Chi, Tai):ti,ab,kw OR (Tai Chi Chuan):ti,ab,kw OR (Taiji):ti,ab,kw OR (Taijiquan):ti,ab,kw OR (T'ai Chi):ti,ab,kw |
| **#18** | (Tai Ji Quan):ti,ab,kw OR (Ji Quan, Ta):ti,ab,kw OR (Quan, Tai Ji):ti,ab,kw OR (Baduanjin):ti,ab,kw OR (Wuqinxi):ti,ab,kw |
| **#19** | (Yijinjing):ti,ab,kw OR (Yoga):ti,ab,kw OR (Exercise Movement Techniques):ti,ab,kw OR (Movement Techniques, Exercise):ti,ab,kw OR (Exercise Movement Technics):ti,ab,kw |
| **#20** | (Pilates-Based Exercises):ti,ab,kw OR (Exercises, Pilates-Based):ti,ab,kw OR (Pilates Based Exercises):ti,ab,kw OR (Pilates Training):ti,ab,kw OR (Training, Pilates):ti,ab,kw |
| **#21** | #14 or #15 or #16 or #17 or #18 or #19 or #20 |
| **#22** | #4 and #13 and #21 |

**Supplementary Table 1** .Research retrieval methods based on Cochrane Library

**Supplementary Table 2** .Research retrieval methods based on PubMed.

| **Steps** | Search Strategies |
| --- | --- |
| **#1** | Students[MeSH Terms] |
| **#2** | **((((((((Students[Title/Abstract]) OR (Student[Title/Abstract])) OR (School Enrollment[Title/Abstract])) OR (Enrollment, School[Title/Abstract])) OR (Enrollments, School[Title/Abstract])) OR (School Enrollments[Title/Abstract])) OR (College students[Title/Abstract])) OR (High school students[Title/Abstract])) OR (Middle school student[Title/Abstract])** |
| **#3** | #1 OR #2 |
| **#4** | ((Anxiety[MeSH Terms]) OR (Depression[MeSH Terms])) OR (Sleep[MeSH Terms]) |
| **#5** | **((((((((((((((((((((((Anxiety[Title/Abstract]) OR (Angst[Title/Abstract])) OR (Nervousness[Title/Abstract])) OR (Hypervigilance[Title/Abstract])) OR (Social Anxiety[Title/Abstract])) OR (Anxieties, Social[Title/Abstract])) OR (Anxiety, Social[Title/Abstract])) OR (Social Anxieties[Title/Abstract])) OR (Anxiousness[Title/Abstract])) OR (Depression[Title/Abstract])) OR (Depressive Symptoms[Title/Abstract])) OR (Depressive Symptom[Title/Abstract])) OR (Symptom, Depressive[Title/Abstract])) OR (Emotional Depression[Title/Abstract])) OR (Depression, Emotional[Title/Abstract])) OR (Sleep[Title/Abstract])) OR (Sleep Habits[Title/Abstract])) OR (Habit, Sleep[Title/Abstract])) OR (Habits, Sleep[Title/Abstract])) OR (Sleep Habit[Title/Abstract])) OR (Sleeping Habit[Title/Abstract])) OR (Habit, Sleeping[Title/Abstract])) OR (Habits, Sleeping[Title/Abstract])** |
| **#6** | #4 OR # 5 |
| **#7** | **Mind-Body Therapies[MeSH Terms]** |
| **#8** | **(((((((((((((((((((((((((((((Mind-Body Therapies[Title/Abstract]) OR (Mind Body Therapies[Title/Abstract])) OR (Mind-Body Therapy[Title/Abstract])) OR (Therapies, Mind-Body[Title/Abstract])) OR (Therapy, Mind-Body[Title/Abstract])) OR (Mind-Body Medicine[Title/Abstract])) OR (Mind Body Medicine[Title/Abstract])) OR (Tai Ji[Title/Abstract])) OR (Tai-ji[Title/Abstract])) OR (Tai Chi[Title/Abstract])) OR (Tai Chi Chuan[Title/Abstract])) OR (Taiji[Title/Abstract])) OR (Chi, Tai[Title/Abstract])) OR (Taijiquan[Title/Abstract])) OR (T'ai Chi[Title/Abstract])) OR (Tai Ji Quan[Title/Abstract])) OR (Ji Quan, Tai[Title/Abstract])) OR (Quan, Tai Ji[Title/Abstract])) OR (Baduanjin[Title/Abstract])) OR (Wuqinxi[Title/Abstract])) OR (Yijinjing[Title/Abstract])) OR (Yoga[Title/Abstract])) OR (Exercise Movement Techniques[Title/Abstract])) OR (Movement Techniques, Exercise[Title/Abstract])) OR (Exercise Movement Technics[Title/Abstract])) OR (Pilates-Based Exercises[Title/Abstract])) OR (Exercises, Pilates-Based[Title/Abstract])) OR (Pilates Based Exercises[Title/Abstract])) OR (Pilates Training[Title/Abstract])) OR (Training, Pilates[Title/Abstract])** |
| **#9** | #7 OR#8 |
| **#10** | #3 AND #6 AND #9 |

**Supplementary Table 3** .Research retrieval methods based on Embase

| **Steps** | Search Strategies |
| --- | --- |
| **#1** | 'student'/exp |
| **#2** | 'students':ab,ti |
| **#3** | 'student':ab,ti |
| **#4** | 'school enrollment':ab,ti |
| **#5** | 'enrollment, school':ab,ti |
| **#6** | 'enrollments, school':ab,ti |
| **#7** | 'school enrollments':ab,ti |
| **#8** | 'college students':ab,ti |
| **#9** | 'high school students':ab,ti |
| **#10** | 'middle school student':ab,ti |
| **#11** | #1 OR #2 OR #3 OR #4 OR #5 OR #6 OR #7 OR #8 OR #9 OR #10 |
| **#12** | 'anxiety'/exp |
| **#13** | 'depression'/exp |
| **#14** | 'sleep'/exp |
| **#15** | 'anxiety':ab,ti |
| **#16** | 'angst':ab,ti |
| **#17** | 'nervousness':ab,ti |
| **#18** | 'hypervigilance':ab,ti |
| **#19** | 'social anxiety':ab,ti |
| **#20** | 'anxieties, social':ab,ti |
| **#21** | 'anxiety, social':ab,ti |
| **#22** | 'social anxieties':ab,ti |
| **#23** | 'anxiousness':ab,ti |
| **#24** | 'depression':ab,ti |
| **#25** | 'depressive symptoms':ab,ti |
| **#26** | 'depressive symptom':ab,ti |
| **#27** | 'symptom, depressive':ab,ti |
| **#28** | 'emotional depression':ab,ti |
| **#29** | 'depression, emotional':ab,ti |
| **#30** | 'sleep':ab,ti |
| **#31** | 'sleeping habits':ab,ti |
| **#32** | 'sleep habits':ab,ti |
| **#33** | 'habit, sleep':ab,ti |
| **#34** | 'habits, sleep':ab,ti |
| **#35** | 'sleep habit':ab,ti |
| **#36** | 'sleeping habit':ab,ti |
| **#37** | 'habit, sleeping':ab,ti |
| **#38** | 'habits, sleeping':ab,ti |
| **#39** | #12 OR #13 OR #14 OR #15 OR #16 OR #17 OR #18 OR #19 OR #20 OR #21 OR #22 OR #23 OR #24 OR #25 OR #26 OR #27 OR #28 OR #29 OR #30 OR #31 OR #32 OR #33 OR #34 OR #35 OR #36 OR #37 OR #38 |
| **#40** | 'mind-body therapies':ab,ti |
| **#41** | 'mind body therapies':ab,ti |
| **#42** | 'mind-body therapy':ab,ti |
| **#43** | 'therapies, mind-body':ab,ti |
| **#44** | 'therapy, mind-body':ab,ti |
| **#45** | 'mind-body medicine':ab,ti |
| **#46** | 'mind body medicine':ab,ti |
| **#47** | 'tai ji':ab,ti |
| **#48** | 'tai-ji':ab,ti |
| **#49** | 'tai chi':ab,ti |
| **#50** | 'chi, tai':ab,ti |
| **#51** | 'tai chi chuan':ab,ti |
| **#52** | 'taiji':ab,ti |
| **#53** | 'taijiquan':ab,ti |
| **#54** | 'tai chi':ab,ti |
| **#55** | 'tai ji quan':ab,ti |
| **#56** | 'ji quan, tai':ab,ti |
| **#57** | 'quan, tai ji':ab,ti |
| **#58** | 'baduanjin':ab,ti |
| **#59** | 'wuqinxi':ab,ti |
| **#60** | 'yijinjing':ab,ti |
| **#61** | 'yoga':ab,ti |
| **#62** | 'exercise movement techniques':ab,ti |
| **#63** | 'movement techniques, exercise':ab,ti |
| **#64** | 'exercise movement technics':ab,ti |
| **#65** | 'pilates-based exercises':ab,ti |
| **#66** | 'exercises, pilates-based':ab,ti |
| **#67** | 'pilates based exercises':ab,ti |
| **#68** | 'pilates training':ab,ti |
| **#69** | 'training, pilates':ab,ti |
| **#70** | #40 OR #41 OR #42 OR #43 OR #44 OR #45 OR #46 OR #47 OR #48 OR #49 OR #50 OR #51 OR #52 OR #53 OR #54 OR #55 OR #56 OR #57 OR #58 OR #59 OR #60 OR #61 OR #62 OR #63 OR #64 OR #65 OR #66 OR #67 OR #68 OR #69 |
| **#71** | #11 AND #39 AND #70 |

**Supplementary Table 4** .Research retrieval methods based on Web of Science

| Steps | Search Strategies |
| --- | --- |
| #1 | TS=(Students) OR TS=(Student) OR TS=(School Enrollment) OR TS=(Enrollment, School) OR TS=(Enrollments, School) OR TS=(School Enrollments) OR TS=(College students) OR TS=(High school students) OR TS=(Middle school student) and Preprint Citation Index (Exclude – Database) |
| #2 | TS=(Anxiety) OR TS=(Angst) OR TS=(Nervousness) OR TS=(Hypervigilance) OR TS=(Social Anxiety) OR TS=(Anxieties, Social) OR TS=(Anxiety, Social) OR TS=(Social Anxieties) OR TS=(Anxiousness) OR TS=(Depression) OR TS=(Depressive Symptoms) OR TS=(Depressive Symptom) OR TS=(Symptom, Depressive) OR TS=(Emotional Depression) OR TS=(Depression, Emotional) OR TS=(Sleep) OR TS=(Sleeping Habits) OR TS=(Sleep Habits) OR TS=(Habit, Sleep) OR TS=(Habits, Sleep) OR TS=(Sleep Habit) OR TS=(Sleeping Habit) OR TS=(Habit, Sleeping) OR TS=(Habits, Sleeping) and Preprint Citation Index (Exclude – Database) |
| #3 | TS=(Mind-Body Therapies) OR TS=(Mind Body Therapies) OR TS=(Mind-Body Therapy) OR TS=(Therapies, Mind-Body) OR TS=(Therapy, Mind-Body) OR TS=(Mind-Body Medicine) OR TS=(Mind Body Medicine) OR TS=(Tai Ji) OR TS=(Tai-ji) OR TS=(Tai Chi) OR TS=(Chi, Tai) OR TS=(Tai Chi Chuan) OR TS=(Taiji) OR TS=(Taijiquan) OR TS=(T'ai Chi) OR TS=(Tai Ji Quan) OR TS=(Ji Quan, Tai) OR TS=(Quan, Tai Ji) OR TS=(Baduanjin) OR TS=(Wuqinxi) OR TS=(Yijinjing) OR TS=(Yoga) OR TS=(Exercise Movement Techniques) OR TS=(Movement Techniques, Exercise) OR TS=(Exercise Movement Technics) OR TS=(Pilates-Based Exercises) OR TS=(Exercises, Pilates-Based) OR TS=(Pilates Based Exercises) OR TS=(Pilates Training) OR TS=(Training, Pilates) and Preprint Citation Index (Exclude – Database) |
| #4 | #3 AND #2 AND #1 and Preprint Citation Index (Exclude – Database) |

**Supplementary Table 5**. Research retrieval methods based on VIP Database and China National Knowledge Infrastructure (CNKI)

| Search Strategies | (((题名或关键词=大学生 OR 题名或关键词=高校学生 OR 题名或关键词=高中生) AND (((((题名或关键词=抑郁 OR 题名或关键词=焦虑) OR 题名或关键词=情志异常) OR 题名或关键词=抑郁症状) OR 题名或关键词=抑郁情绪) OR 题名或关键词=睡眠)) AND ((((((题名或关键词=八段锦 OR 题名或关键词=太极) OR 题名或关键词=气功) OR 题名或关键词=易筋经) OR 题名或关键词=五禽戏) OR 题名或关键词=瑜伽) OR 题名或关键词=普拉提)) |
| --- | --- |

**Supplementary Table 6.** Research retrieval methods based on Wan fang Data

| Search Strategies | 题名或关键词:(大学生 OR 高校学生 OR 高中生) and 题名或关键词:(抑郁 OR 焦虑 OR 情志异常 OR 抑郁症状 OR 抑郁情绪 OR 睡眠) and 题名或关键词:(八段锦 OR 太极 OR 气功 OR 易筋经 OR 五禽戏 OR 瑜伽 OR 普拉提) |
| --- | --- |

**Supplementary Table 7. Consistency test results**

| Outcomes | Consistency test | Inconsistency test | I2(%) |
| --- | --- | --- | --- |
| Depression | 96.55 | 96.37 | 34 |
| Anxiety | 90.45 | 90.44 | 6 |
| Sleep Quality | 34.47 | 34.51 | 4 |

**Supplementary Table 8. The quality of evidence (GRADE) of mind-body therapies**

| Outcomes | Grade |
| --- | --- |
| depression | moderate |
| anxiety | moderate |
| sleep quality | Low |
