# Supplementary figures and images for: Effect of mind–body therapies on anxiety, depression and sleep quality in college students: a network meta-analysis
Source: Front Public Health. 2026 Mar 19;14:1767300. doi: 10.3389/fpubh.2026.1767300 (PMC13043389; doi:10.3389/fpubh.2026.1767300)

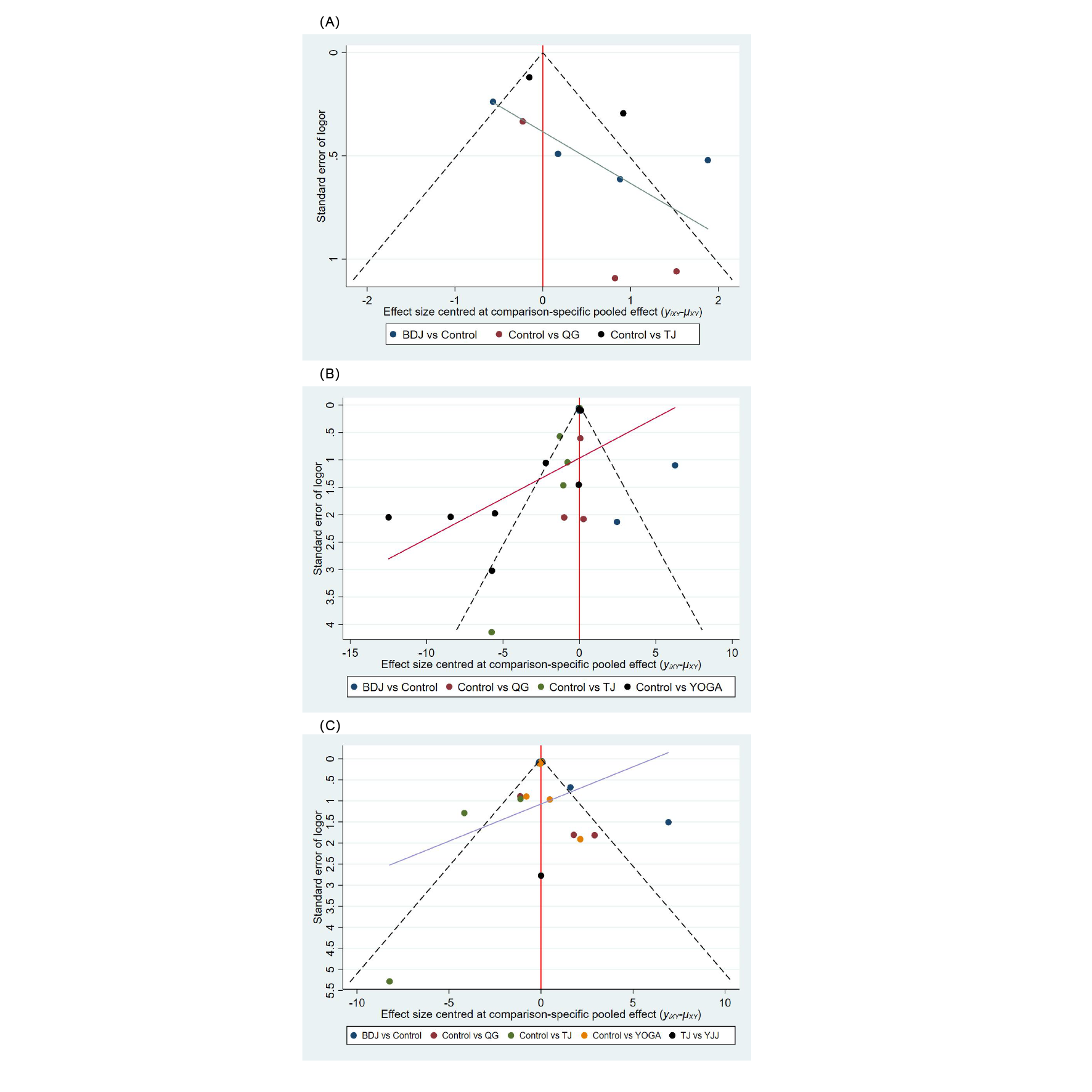

Supplement: Supplementary file 2 [file Image_1.tif]
